# A comparison of surface roughness after micro abrasion of enamel with and without using CPP-ACP: An *in vitro* study

**DOI:** 10.4103/0972-0707.53337

**Published:** 2009

**Authors:** Jones Mathias, S Kavitha, S Mahalaxmi

**Affiliations:** Department of Conservative Dentistry and Endodontics, SRM Dental College, Chennai, India

**Keywords:** CPP-ACP, micro abrasion, surface roughness

## Abstract

**Aim:**

The aim of this study was to evaluate the surface roughness of enamel after micro abrasion with and without using remineralization agent, CPP-ACP (Casein Phosphopeptide-Amorphous Calcium Phosphate).

**Materials and Methods:**

Thirty freshly extracted anterior teeth were collected. The samples were randomly assigned to two study and one control group. Group A (n = 10) containing teeth in which only micro abrasion was done, Group B (n = 10) containing teeth in which CPP-ACP (G C Tooth Mousse) was applied after micro abrasion for a period of 30 days, once daily for three minutes and Group C (n = 10) in which no preparation was done and which acted as the control group. The samples were stored in artificial saliva and evaluated after 30 days, using surface profilometer. The results were tabulated and statistically analyzed.

**Results:**

According to the results of this study, a combination of the micro abrasion procedure and CPP-ACP application reduced the enamel surface roughness significantly, when compared to micro abrasion done alone.

**Conclusion:**

Application of CPP-ACP after micro abrasion procedure significantly reduces the enamel surface roughness thereby decreasing the risk of caries.

## INTRODUCTION

Enamel micro abrasion was developed to improve surface texture, remove superficial intrinsic stains, and repair enamel decalcification and texture defects.[1,3] Enamel micro abrasion creates a highly polished prismless (abrosion effect), mineral rich surface, such that it takes a longer time for an acquired pellicle and, subsequently, mutans streptococci, to colonize the smooth surface.[4] Micro abraded surface becomes smooth and lustrous and maintains a glass-like sheen.[4,5] However, although micro abrasion produces a smooth and lustrous surface, it may create a microscopic roughness since hydrochloric acid (HCl) is used in the micro abrasion procedure. As a result of this, it is apparent that in spite of the external appearance, the enamel tends to be porous.

The importance of mutans streptococci in the etiology of dental caries has been recognized since the 1970s. The survival of mutans streptococci in the oral environment depends on their ability to adhere to a surface.[6] Retentive areas of solid tooth surfaces are the preferential colonization sites for mutans streptococci and their presence at high levels is an indicator of increased risk of caries. 

Bollen * et al.* defined the critical roughness threshold beyond which bacteria were likely to adhere to the surface as 0.2*µ*m.[7] Given the nature of the bacteria for adherence, it is sensible to apply preventive strategies based on modification, suppression and elimination of mutans streptococci and thus present demineralization. 

Hence, in this study, micro abrasion followed by application of Casein Phosphopeptide-Amorphous Calcium Phosphate, which is a water based cream, was used to modify the enamel surface,[8,9] after which the surface roughness of enamel was evaluated.

## MATERIALS AND METHODS

- Thirty extracted human anterior teeth
- 11% HCl and finely powdered pumice-slurry
- 10% CPP-ACP (GC Tooth Mousse)
- Artificial saliva
- 0.1% Thymol solution
- Micromotor handpiece
- Rubber cup
- Surface profilometer (Mitutoyo SJ 400)

Thirty freshly extracted anterior teeth, which had no restoration and no carious lesion, were chosen for this study. Teeth which had defects on the enamel surface, erosion, microcracks, and visible stains on the lingual and facial surface were not taken up for this study. Samples were collected and stored in 0.1% thymol solution for three weeks, in order to avoid any fungal or bacterial growth. The teeth were thoroughly cleaned off its debris and soft tissue using an ultrasonic scaler, taking into consideration the fact that only the cervical third of the tooth would be used. The samples were randomly assigned to two experimental and one control group, containing 10 samples each. Group A consisted of teeth in which only micro abrasion was done. Group B consisted of teeth in which 10% CPP-ACP was applied after micro abrasion, for a period of 30 days, once daily for three minutes. Group C consisted of teeth in which no preparation was done and which acted as the control group. All the samples were stored in artificial saliva for a period of thirty days.

## Micro abrasion procedure

A custom made abrasive slurry was prepared with 11% HCl acid and fine powdered pumice.[1,2,10,11] The prepared slurry was applied to the labial surface of the tooth, using a rotating rubber cup. Ten 10- second applications with 20g of pressure were given. The slurry was rinsed away after each application.[12] 

## Application of CPP-ACP

CPP-ACP was applied directly with a clean finger, on the labial surface of the tooth, and smeared over the surface for three minutes.[13,14]

## Roughness measurement

**Figure 1:**
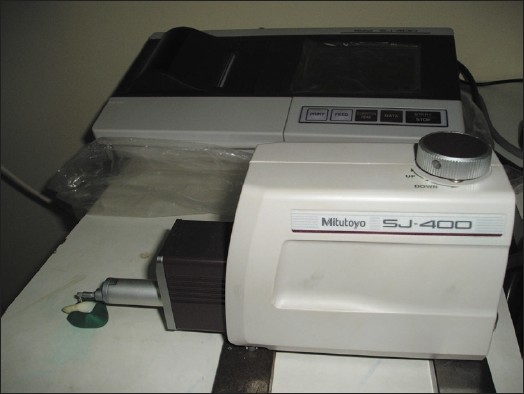
Profilometer-Mitutoyo SJ 400

The method used was to scan a diamond stylus across the surface (5 mm) under a constant load and compute the numeric values representing the roughness of the profile as Ra. The Ra value describes the overall roughness of a surface and is defined as the arithmetic mean value of all absolute distances of the roughness profiles, from the center line within the measuring length. In this study, Ra values were obtained using a Mitutoyo-SJ 400. A diamond stylus (5 *µ*m tip radius) was used under a constant pressure of 3.9 mN Figure 1.[15,16]

## RESULTS

Mean and standard deviation were estimated from the sample for each study group [Tables 1 and 2]. Mean values were compared between different groups by using one-way Anova, followed by Tukey-HSD procedure. In the present study, * P* < 0.05 was considered the level of significance.

**Figure 2:**
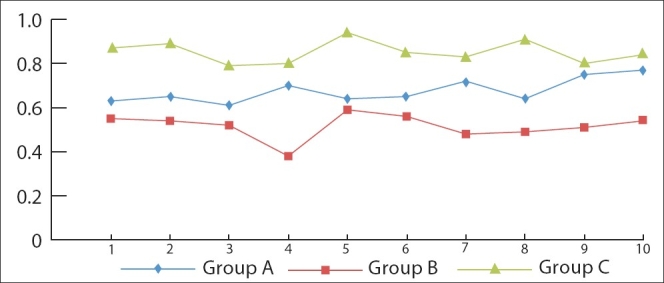
Mean surface roughness

The mean surface roughness value in Group B (samples with micro abrasion procedure and CPP-ACP application) (0.52 ± 0.057) was significantly lower than the mean values in Group-A (samples with micro abrasion procedure only) (0.68 ± 0.055) and Group C (control group) (0.85 ± 0.050) (* P* < 0.005). Further, the mean value in Group A (0.68 ± 0.055) was significantly lower than the mean value in Group C (0.85 ± 0.050) (* P* < 0.005) [Figure 2].

**Table 1:** Surface roughness (*µ*m)

Group-A	Group-B	Group-C
0.63	0.55	0.87
0.65	0.54	0.89
0.61	0.52	0.79
0.70	0.38	0.80
0.64	0.59	0.94
0.65	0.56	0.85
0.72	0.48	0.83
0.64	0.49	0.91
0.75	0.51	0.80
0.77	0.54	0.84

**Table 2:** Mean surface roughness

Group	Mean ± SD	*P*-value	Significant groups at 5% level
A	0.68 + 0.055	< 0.0001 (Sig)	C vs. A, B
B	0.52 + 0.057		A vs. B
C	0.85 + 0.050

## DISCUSION

The enamel surface structure is composed of hydroxyapatite and each crystal of Ca_10_ (PO_4_)6(OH)_2_ is surrounded by a layer of tightly bound water. The presence of this hydration shell shows that the crystal is electrically charged and can therefore attract ions that are able to play a part in remineralization. As a result of the above, it is apparent that, in spite of the external appearances, enamel is porous and ion migration is possible.[17] 

Micro abrasion is a procedure normally followed to remove surface discoloration. It was claimed that this procedure produced a smooth surface. However, during the micro abrasion procedure, the acid used can penetrate enamel and the ions can be displaced. Therefore, there is a possibility of increasing the level of porosity, thus facilitating acid transport and further demineralization. However, it is apparent that in the enamel surface, the opposite action is also possible, with the ions returning along the same pathways, so that the enamel can remineralize. Hence, in this study CPP-ACP was used.

* Mutans streptococci* , besides being acidogenic and aciduric, need to adhere to the surface to initiate dental caries. Basically, every surface has a critical roughness threshold beyond which bacteria are likely to adhere to the surface and the value of this threshold is 0.2*µ*m.[7] The study of tooth surface roughness thus becomes very important, because it is a reflection of the adherence of bacteria, which is the prime source for the initiation of dental caries.

According to the results of this study, a combination of micro abrasion and ACP-CPP has reduced the enamel surface roughness significantly, when compared to micro abrasion done alone. This may be because CPP-ACP, which are naturally occurring molecules, are able to bind calcium and phosphate ions and stabilize amorphous calcium phosphate in metastable solution.[18] It maintains the pores in enamel surface, which consequently reduces bacterial adherence. When CPP-ACP is applied in the oral environment, it will bind to biofilms, plaque, bacteria, hydroxyapatite and soft tissue, localizing bio available calcium and phosphate ions. It will thus maintain a state of super saturation with respect to tooth enamel, reducing demineralization and enhancing remineralization.[8,9,13,14] Saliva will enhance the effectiveness of CPP-ACP and the flavor will help stimulate saliva flow. Several studies claim that the longer CPP-ACP and saliva are maintained in the mouth, the more effective the result. The surface roughness of micro abraded enamel surface was better, compared to the control group, the untreated surface. This is in accordance with the study done by Seguera.[4] This may be because the micro abrasion procedure creates a highly polished and prismless structure, the abrosion effect, thus creating a smooth surface.

Further investigations need to be done to bring down the surface roughness to less than 0.2 *µ*m, which is the optimal threshold level.[7]

The entire study was done using profilometry, which had an advantage of precisely measuring the surface roughness, without the need for additional quantitative analysis.[15–17]

## CONCLUSIONS

- Under the limitations of this study, 10% CPP-ACP can be applied on enamel surface following micro abrasion, as it reduces the enamel surface roughness, as compared to the micro abrasion procedure when done alone. Thus, application of CPP-ACP can prevent colonization of bacteria.
- Surface profilometric analysis (Mitutoyo SJ400) was found to be an efficient way to quantitatively assess the surface roughness of enamel * in vitro*.
